# The relationship between patriotism and regional identification: a cross-country analysis

**DOI:** 10.1007/s00168-022-01167-1

**Published:** 2022-08-18

**Authors:** Peter Dirksmeier

**Affiliations:** Institute of Economic and Cultural Geography, Leibniz Universität Hannover, Schneiderberg 50, 30167 Hanover, Germany

**Keywords:** Z00, Z19

## Abstract

Patriotism—as an ambivalent attitude towards the nation—has less exclusionary characteristics than nationalism and regional identification, because it does not require comparisons and hierarchies. What is less clear, however, is how to explain the positive evaluation of patriotism in the wider population. The article analyses the positive relationship of patriotism with nationalism and regional identification in 29 national and 421 regional contexts. The paper clearly shows that different factors explain the positive evaluation of patriotism and the mind-set of patriotism itself. While a nationalist attitude and regional identification at the individual level are strongly associated with a positive evaluation of patriotism and patriotism itself, institutionalised forms of regional autonomy are shown to be insignificant for the evaluation of patriotism and ambivalent for patriotism itself at the context level. The article concludes by discussing these results in the context of a Janus-faced nature of regional identification that can contribute to an inclusive society as much as to a nationalist-chauvinist attitude and which has so far been overlooked in regional science.

**Supplementary Information:**

The online version contains supplementary material available at 10.1007/s00168-022-01167-1.

## Introduction

Patriotism can be considered a significant ideational connection of people to a territory. As an attitude, patriotism can be conducive to social cohesion, but it can also be exclusionary towards minorities with other nationalities (Ariely 2018). Patriotism appears predominantly ambivalent. Yet, patriotism is evaluated less negatively than its counterparts nationalism (Macedo 2011) or regional identification. On the other hand, it is noticeable that regional attachment to place receives less attention compared to patriotism. Relatively little work exists in regional science that deals with the mutual influence of patriotism, nationalism, and regional attachment to place as regional identification as well as their impact on further exclusionary and problematic mindsets for society, such as anti-immigrant attitudes or the inclusion of minorities (Agnew 2013; Dirksmeier 2021; Escandell and Ceobanu 2010; Fitjar 2010; Green et al. 2011).

What remains predominantly unnoticed in this discourse about the intersections of regional attitudes towards the national, however, is how people *evaluate* patriotism (Ariely 2018) and, in particular, which role especially regional identification plays in the evaluation of patriotism. Do regionalist attitudes also lead to favouring patriotism? Or is patriotism, including its positive evaluation, a separate outlook that is clearly distinct from regionalist and nationalist attitudes? In particular, the influence of regionalist attitudes on the evaluation of patriotism and patriotism itself has not yet been researched in depth. This research gap seems all the more surprising considering the great importance of forms and repercussions of regional attachments studied by social sciences in a variety of contours (Agnew 2013; Chiang and Jane 2013; Ciută 2008; Glass 2018; Luukkonen and Sirviö 2019; Makarychev and Yatsyk 2018; Sindre 2018).

One possible explanation for this situation could be the unclear territorial reference of the region. Patriotism and nationalism refer to the nation clearly delimited with the Westphalian territorial state. However, it is often overlooked that people do not necessarily feel that they belong to only one nation (Bonikowski 2016). Nations emerged from smaller regional units, which, for example in Germany, were formed via an integration of local loyalties of confessions and political identities in a regional conception of homeland as an inherent component of the new fatherland (Agnew 2018). In contrast, in a relational perspective, the region is defined as a relationship between scale levels, which emerges on the basis of practices and discourses (Paasi et al. 2018), less as a scale level itself (Cochrane 2018). In such a conception of region, however, the boundaries of the territorial units of reference become blurred, but these still are important containers of socialisation and, consequently, produce the discourses and practices that afterwards define regions (Storey 2018). The significance of regions as important actors in human practice can be seen, for example, in the highly differentiated regional voting patterns on Brexit (Agnew 2018) or in the coincidence of regionalist and nationalist aspirations in Scottish nationalism (Smith 1995).

To contain the problem of the relationality of regions in discourse, an older proposal by Jürgen Habermas on constitutional patriotism (Habermas 2019) is used here to operationalise regional identification. For the analysis of patriotism, the conception of region as a territorial entity organised by parliamentary institutions is considered essential (Habermas 2019). Regional identification can therefore be operationalised, in extension of Habermas’ work, as a sense of belonging to the small-scale territorial authorities of place and district, which usually have institutional representation and thus opportunities for political co-determination. With this view, the paper contributes to the understanding of territorial place attachments at the regional scale level for the discourse on relational understandings of region and regional identification (Paasi et al. 2018; Söderbaum 2013; Varró and Lagendijk 2013). It thus adds to social scientific knowledge on regional societies and their “wide range of social and cultural characteristics” (Agnew 2013, 15).

The database for the study is the International Social Survey Programme (ISSP) 2013 (GESIS 2015a) that contains representative samples of the adult population in 34 national states, where Germany is included twice in the sample (East/West). ISSP 2013 includes questions concerning patriotism, national and regional identity as well as on the assessment of patriotism. For measuring the impact of regional identification and nationalism more precisely, first the ISSP data set is complemented with macrodata at the country level concerning the extent of regional authority (Hooghe et al. 2016), the presence of an autonomous region in the respective country taken from the Database of Political Institutions (Cruz et al. 2018), the effective democracy index (Welzel 2015), willingness to fight for the country index (Welzel 2015), and net migration (IOM Global Migration Data Analysis Centre 2019). Second, regional identification, *völkisch* nationalism, and anti-immigrant attitudes variables are aggregated at the regional level and also included. Urban region serves as an additional control variable at the regional level. The paper is structured as follows: first, it outlines the background context of positive evaluation of patriotism, patriotism, and regional identification in detail. The third part introduces the analysis strategy and presents information on the data set. The subsequent section presents the significant results of the regression analyses. The outcomes reveal that regionalist attitudes are related to a positive assessment of patriotism, but that this applies to institutionally guaranteed administrative rights at the regional and country level to a limited extent at best. The paper concludes with a discussion of these empirical findings and draws further implications.

##  Background: patriotism and regional identification

Patriotism represents a positive attitude towards state institutions and achievements expressed through pride in the state and country (Green et al. 2011) and is at the same time the subject of evaluations and value attitudes. Patriotism emerges without comparisons to other countries, and, thus, mostly without hierarchies and negative assessments (de Figueirdo Jr. and Elkins 2003). Patriotism is rather an affective attachment to a specific country that could be described as a form of critical loyalty (Ariely 2011), but is equally discussed in a cosmopolitan perspective (Erez and Laborde 2019). Even when patriotism also arises from a specific peer group (Esses et al. 2005; Green et al. 2011), it only expresses the affective dimension of one’s pride in a nation (Esses et al. 2005). Thus, patriotism is self-referential, free from competition as the semantics of patria imply, and mostly expressed as close identification with the social values and system of a country or group (de Figueirdo Jr and Elkins 2003). Patriotism less reflects peer group interests and more a benign form of national attachment (Esses et al. 2005).

Unlike nationalism in its *völkisch* or chauvinist varieties, patriotism as an attitude is less clearly defined. Patriotism can promote internal cohesion in a society, foster social commitment and solidarity and thus contribute to the integration of immigrants (Ariely 2018). It was pointed out early on that patriotism can only be a value in itself if immigrants can also be patriotic (Stewart 1917). Empirical studies, on the other hand, indicate that immigrants show less pronounced patriotism than autochthones (Ariely 2018). Patriotism as “affective attachment to one’s nation” (Esses et al. 2005, 320) is expressed, for example, in a greater willingness to pay taxes or to participate in political processes (Ariely 2018). What is particularly interesting is the spatial extension of the targets of patriotic sentiment. While in antiquity, these were still strictly local, in modern times, they extend to the national state (Tuan 1974). Nevertheless, patriotism also shows parallels with a heightened and subsequent exclusionary national consciousness (Ariely 2018). Patriotism can be associated with the drawing of boundaries between peer groups, it directly influences how immigrants are appraised (Green et al. 2011). This indifferent position of patriotism becomes clear in the controversy over the distinction between patriotism and nationalism since phenomena assigned to nations overlap in especially “banal” forms of nationalism (Billig 1995) and patriotism in terms of flags (Becker et al. 2017), anthems, passports, sports stars, or pride in one’s country (Webster 2011).

The connection between patriotism and regional identification is much less researched than the relationship between patriotism and nationalism (Heinrich 2020). In current geographical and regional research, the very theoretical status of the region is hotly disputed. The regional scale has become a significant political competitor for the territorially framed nation state, as the Brexit vote or votes in Catalonia show (Agnew 2018). Regions thus intervene in political processes. Geographical discourse nevertheless debates the region in depth as if it did not seem to know any defined borders (Agnew 2018), and this happens although regional inequality is accepted as a historical constant (Massey 1979). Regions are “active processes rather than fixed categories” (Cochrane 2018, 82). They are social constructs whose emergence owes much to social practices and discourses. Thus, regions are always also (local) reactions to capitalist processes and ultimately a medium of interaction between action and structure (Paasi et al. 2018). This local response to macroeconomic conditions is responsible for a region gaining its concrete form, because of the local structures of feelings that are supposed to “hold together” regional spaces in their relationality (Paasi 2010). As Milner (1994) emphasises, structures of feeling can be understood as a “generation-specific medium of processing human experiences” (Dirksmeier 2016, 889). Region, from the relational perspective, is thus a process of sedimentation of an emerging region into the spatial structure and spatial consciousness of a society (Varró and Lagendijk 2013), or even local community.

The relational conception of region, however, is very difficult to operationalise. Regional identities are very much capable of confronting nation states with the evolutionary step of political rescaling up to secession (Calzada 2018). This can only be stated from a relational perspective. The empirical analysis of regional identification, however, faces the problem that these attachments are, in the relational view, not tied to a territory but to discursive processes that cannot be easily translated into empirical research. Furthermore, Varró and Lagendijk (2013) criticise that the relational conceptualisations of regions do not always consistently refer to the basic poststructuralist assumptions. Rather, this kind of poststructuralist argumentation is characterised by “a symbiosis of incompatibles […], an amalgam that at its core resists ‘normal’ scientific analysis”, as Jürgen Habermas (1988, 390; own translation) criticises. The problem is the dominance of theories of regionalism that translate European experiences into a language with an affinity for theory (Söderbaum 2013).

The current direction of discourse on regions points away from singular understandings of region and territory and towards conscious plurality. Paasi et al. (2018) use the term *comparative regionalism,* “by this we mean, establishing mechanisms and networks, which promote greater engagement across contexts and territories” (Paasi et al. 2018, 17). Early works on comparative regionalism (Söderbaum 2013; 2016) focus in particular on a regional identification, which in the previous discourse was merely contrasted with a misplaced universalism. A conceptual sharpening of the understanding of regions as open entities that extend in space could be achieved if more work were done on the regional bonding of people. This regional identification or regionalism is one of the important mechanisms of region making cited by Paasi et al. (2018). In the context of his analysis of constitutional patriotism, Jürgen Habermas (2019) proposes to first focus on small-scale regions that are empirically tangible and at the same time real in lifeworld terms. This opens up the possibility of an empirical analysis of regional identification, without at the same time falling into the territorial reduction trap (Varró and Lagendijk 2013). The region is then to be understood as a practice, similar to what progressive works are trying to do with territory (Blomley 2016). With that said, the imaginings of the respective group that feels associated with the specific small segment of space constituting the region, be it as a sense of place, a structure of feeling, or a sense of home, are, thus, the main sphere of interest for social science efforts at researching regional identification in its relation to patriotism. As Escandell and Ceonbanu (2008) point out, patriotism can exist in a blind and a constructive form and, thus, in the latter case be diametrically opposed to *völkisch* or chauvinist notions of nationalism. At this point, the role of regional identification, although also referring to a cognitive relational unit with a spatial equivalent, remains unclear in the literature. Following these insights, a regionalist attitude should have a promoting effect on the evaluation of patriotism and patriotism itself [Hypothesis 1]. If regional identification promotes patriotic attitudes and positive evaluations of patriotism, then a higher degree of regional autonomy should also have a positive effect on the evaluation of patriotism as well as the patriotism attitude [Hypothesis 2].

## Data and analysis strategy

As the data structure has three levels and consists of individuals living in subnational regions in nation states with varying degrees of regional autonomy, linear multilevel models based on maximum likelihood estimations as the main regression technique are calculated in order to include the assumed context effects of the national and regional level. Two baseline models are used to test the proportion of explained variance for the dependent variables *evaluation of patriotism* and *patriotism*, which is due to the difference in group contexts. The baseline model is specified as

$${Y}_{ijk}= {\beta }_{0}+{v}_{0k}+{u}_{0jk }+{\epsilon }_{ijk}$$

where ${\beta }_{0}$ is the intercept of the regression, i.e. the mean of ${Y}_{ijk}$ in the sample. ${v}_{0k}$ denotes the variance of the errors at the highest level, ${u}_{0jk}$ at the regional level, and ${\epsilon }_{ijk}$ at the individual level (Hox et al. 2018). The variance of the individual-level error term is estimated in parallel with the variance of the country-specific and region-specific error components, which allows us to calculate the proportion of variance attributable to between-context differences (Weins 2011). The interclass correlations clarify that 10.3% of the variance in the patriotism rating scale can be traced back to differences between the countries and 3.9% to differences between the regions. Similarly, 25.1% of the variance in the patriotism scale can be attributed to country differences and 3.4% to regional differences. The correct number of random slopes in multilevel regression is debated controversially (Heisig et al. 2017; Schmidt-Catran and Fairbrother 2016). Yet, the hypothesis that regionalism influences the evaluation of patriotism at the individual level suggests variability in slopes between contexts for the individual latent predictors of regional identification and nationalism. For this reason, random coefficient models are estimated according to the general formula:

$${Y}_{ijk}= {\beta }_{0j}+ {\beta }_{1}{\mathrm{\rm X}}_{ijk}+ {\beta }_{2}{\mathrm{\rm Z}}_{jk}+ {\beta }_{3}{\mathrm{\rm X}}_{ijk}{\mathrm{\rm Z}}_{jk}+ {u}_{1jk}{\mathrm{\rm X}}_{ijk}+ {v}_{0k}+ {u}_{0jk}+ {\epsilon }_{ijk}$$

To meet the minimum standard formulated in this discourse for a balance between flexibility and economy in the models (Heisig et al. 2017), random slopes were included for chauvinistic nationalism, as well as for regional identification. Since the number of countries in the sample is relatively small in contrast to the regions, a robustness test with restricted maximum likelihood with Kenward–Roger Approximation for optimising significance tests of fixed effects (Kenward and Roger 1997) is also calculated (Supplement 1). The results are similar to those of the maximum likelihood method.

The study is based on the International Social Survey Programme—National Identity III (ISSP). The ISSP has been conducted since 1985 as a collaborative cross-national survey programme, using simple or multi-stage stratified random samples of the adult populations and containing 45,297 individuals nested in 33 countries (GESIS 2015a). The sample sizes vary between 904 for the United Kingdom and 2,739 for South Africa. The individual data were supplemented with country-level data on regional authorities that have “the capacity to make legitimate and binding decisions for a collectivity” according to Hooghe et al. (2016, 16) and the presence of an autonomous region in the respective country derived from the database of political institutions 2017 (Cruz et al. 2018) as measures of the respective degree of regional autonomy. Two indexes of the effectiveness of democracy and the willingness to fight for the country according to Welzel (2015), as well as the net migration rate in 2015 from the IOM (https://migrationdata.org), are used as controls. There is no value for the Regional Authority Index for South Africa, Taiwan, Georgia, and India. These states were therefore removed from the sample. Originally, Germany was included separately as East and West Germany in the raw data. Since Germany has been reunified for over 30 years, these data have been combined. The final data set consists of 36,919 individuals nested in 29 state contexts with 421 regions.

The dependent variable is the individual-level positive evaluation of patriotism and patriotism as an attitude. The ISSP data set includes four questions concerning a positive or negative evaluation of patriotism in the respective country. The two positive ratings, namely “How much do you agree or disagree that strong patriotic feelings in [COUNTRY] strengthen [COUNTRY’s] place in the world” and “How much do you agree or disagree that strong patriotic feelings in [COUNTRY] are needed for [COUNTRY] to remain united” (GESIS 2015b), were condensed into one scale (*α* = 0.77). Patriotism is constructed as a scale (*α* = 0.73) from the sum of three items concerning pride in the functioning of democracy, the social welfare system, and the fair treatment of all social groups, which ranges from 1 (not proud at all) to 4 (very proud). Table 1 shows the average values of the dependent variables for the states as well as the number of regions in the respective countries in the sample.

**Table 1:** Distribution of the dependent variables

State	Number of respondents	Number of regions	Evaluation of patriotism (mean/*SD*)	Patriotism (mean/SD)
Belgium	2,100	11	7.55/1.69	8.46/1.83
Croatia	994	6	6.89/2.12	6.17/2.03
Czech Republic	1,894	14	7.16/1.82	6.18/1.96
Denmark	1,317	5	6.97/2.20	8.81/1.75
Estonia	993	5	7.58/1.74	6.18/1.95
Finland	1,221	19	6.77/1.84	8.56/1.88
France	1,982	95	7.39/1.98	8.19/1.91
Germany	1,714	17^a^	6.69/1.77	8.34/1.71
Hungary	994	8	7.07/1.78	6.74/2.09
Iceland	1,055	8	6.94/1.72	7.65/1.75
Ireland	1,167	8	6.85/1.75	7.30/2.02
Israel	1,169	5	7.66/1.83	7.13/2.08
Japan	1,212	9	7.51/1.81	8.15/1.84
Korea (south)	1,294	13	8.39/1.36	7.11/1.80
Latvia	1,000	6	7.72/1.82	5.65/2.01
Lithuania	1,190	10	7.24/1.54	6.30/1.77
Mexico	996	16	7.97/1.72	5.65/2.25
Norway	1,532	6	6.49/1.72	9.28/1.66
Philippines	1,199	4	8.54/1.34	8.43/2.04
Portugal	995	5	7.52/1.49	6.02/1.87
Russia	1,498	11	7.85/1.67	6.06/2.24
Slovak Republic	1,152	8	7.55/1.76	6.38/1.96
Slovenia	1,006	12	7.32/1.60	5.74/1.61
Spain	1,223	17	6.06/2.42	7.16/2.22
Sweden	1,050	69	6.09/1.84	8.30/1.79
Switzerland	1,233	7	6.65/1.75	9.52/1.55
Turkey	1,615	12	8.55/1.67	8.01/2.74
United Kingdom	861	6	7.37/1.63	8.52/1.80
United States	1,263	9	8.05/1.58	8.29/1.86

Source: GESIS 2015a

^a^Berlin is included in the sample separately for East and West Berlin

With nationalism and regional identification, two different concepts of attachment to place and territory are added. Nationalism in the chauvinist variant subsumes three items representing a superior position of one’s own nation (*α* = 0.69), namely “I would rather be a citizen of [COUNTRY] than of any other country in the world”, “The world would be a better place if people from other countries were more like the [COUNTRY NATIONALITY]”, and “Generally speaking, [COUNTRY] is a better country than most other countries” (GESIS 2015b). The *völkisch* version of nationalism, which is clearly different from chauvinistic nationalism (Pehrson et al. 2009), is the sum of two variables: “How important do you think each of the following is: (1) to have been born in [COUNTRY]? (2) to have [COUNTRY NATIONALITY] ancestry?” (GESIS 2015b). The answer could be given on a five-point scale. (*α* = 0.73). Since 23.7% of the answers to these two questions are missing, the variable was aggregated and only included at the regional level. Regional identification subsumes the two items (*α* = 0.76) “How close do you feel to your town or city?” and “How close do you feel to your county?” ranging on a four-point answer scale. The three variables were calculated as the sum of respective items and standardised by calculating the *z*-scores across all countries. They were also aggregated at the regional scale. For urban regions, the variable values for big city and suburbs were first combined and coded with 1 for “metropolitan”, all others with 0. The percentage values for “metropolitan” per region were then *z*-transformed and form the value for urban regions on the region level. The data include eight questions concerning attitudes towards immigrants that range on a five-item scale from “agree strongly” to “disagree strongly”. The answers are recoded so that high figures represent greater rejection of immigrants. Anti-immigrant sentiment is calculated as the sum of these eight items (*α* = 0.79) and aggregated at the regional scale.

At the individual level, sex is indicated by 0 (female) and 1 (male). Age was *z*-transformed, and missing cases were imputed using regression imputation (0.3% of values imputed) (Baltes-Götz 2013). Education is recoded into ‘years in formal education’, where missing values are also imputed with the regression method (1.7% of values imputed) (Baltes-Götz 2013). For instance, the level “no formal education” was recoded as zero years of school education and the level “still in college” was recoded as 14 following Weins (2011). Immigration background is indicated by 0 (yes) and 1 (no) as individual migration history is a strong predictor for weaker patriotism (Ariely 2018). Table 2 shows the descriptive statistics.

**Table 2:** Descriptive statistics

Variable	Definition	Data source	Mean	SD	Min	Max	Frequency
*Dependent variables*							
Evaluation of patriotism	Two-item scale (*α* = 0.77)	ISSP	7.35	1.89	2	10	33,332
Patriotism	Three-item scale (*α* = 0.73)	ISSP	7.47	2.27	3	12	32,146
*Individual level*							
Sex	(1 = male)	ISSP	0.47	0.50	0	1	36,919
Age	in years (imp.)	ISSP	47.86	17.4	10	112	36,919
Education	years of schooling (imp.)	ISSP	12.57	4.2	0	76	36,919
Immigration background	self and/or parents (1 = no)	ISSP	0.88	0.32	0	1	36,919
Regional identification	Two-item scale (*α* = 0.76)	ISSP	6.11	1.46	2	8	35,664
Nationalism (chauvinistic)	Three-item scale (*α* = 0.69)	ISSP	10.22	2.57	3	15	33,752
*Context level*							
Regional authority index	(*z*-score)	Hooghe et al. (2016)	0.00	1.01	− 1.24	2.12	29
Autonomous region	(1 = yes)	Cruz et al. (2018)	0.35	0.48	0	1	29
Effective democracy index		WVS, Welzel (2015)	0.67	0.24	0.12	0.97	29
Willingness to fight for country index	(1 = yes)	WVS, Welzel (2015)	0.66	0.16	0.28	0.89	29
Net migration rate 2015		IOM	1.16	4.13	− 9.7	9.8	29
*Regional level*							
Regional identification	Aggregated	ISSP	6.11	0.47	3	7.87	421
Nationalism (völkisch)	Aggregated	ISSP	7.10	0.73	3	9	421
Anti-immigrant sentiment	Aggregated	ISSP	24.11	2.26	10	34	421
Urban region	Aggregated	ISSP	42.30	28.73	0	100	421

All variables (except dummies) are included as *z*-scores in the regression analyses

Sources: Cruz et al. (2018); GESIS (2015a); Hooghe et al. (2016); IOM Global Migration Data Analysis Centre (2019); Welzel (2015)

A confirmatory factor analysis tests the independence of the items evaluation of patriotism, patriotism itself, chauvinistic nationalism and regional identification from each other following Mußotter (2021). The test results are acceptable (*Χ*^2^ = 1968.016; df = 29; *p* < 0.001; CFI = 0.985; TLI = 0.977; SRMR = 0.036; RMSEA = 0.049) with factor loadings between 0.63 and 0.91. As the scales are sums of ordinal items, the WLS estimator is used to achieve the best possible estimates (Mußotter 2021). The confirmatory factor analysis confirms that the four territory-based attitude scales are independent of each other. The measurement scales can therefore be considered as valid (Mußotter 2021).

## Results and discussion

A total of five multi-level models were calculated. In addition to the baseline model, a control, individual, and regional model, as well as an overall model, are included in the analysis. First, Table 3 presents the results of the baseline, control, and individual models.

**Table 3:** Baseline, control, and individual models

	Model (0)		Model (1)		Model (2)		
	Baseline		Control		Individual		
	Evaluation	Patriotism	Evaluation	Patriotism	Evaluation		Patriotism
	β/(SE)	β/(SE)	β/(SE)	β/(SE)	β/(SE)		β/(SE)
*Individual level*							
Sex (1 = male)			0.0196	0.0169	0.0065		0.0134
			(0.0103)	(0.0095)	(0.0100)		(0.0092)
Age (*z*-score)			0.0475***	0.0258***	− 0.0143**		− 0.0205***
			(0.0055)	(0.0051)	(0.0054)		(0.0050)
Education (*z*-score)			− 0.1085***	0.0070	− 0.0681***		0.0386***
			(0.0062)	(0.0056)	(0.0061)		(0.0056)
Immigration background (1 = no)			0.0766***	− 0.0819***	0.0199		− 0.1333***
			(0.0179)	(0.0164)	(0.0176)		(0.0162)
Nationalism (chauv.) (*z*-score)					0.3541***		0.2813***
					(0.0346)		(0.0502)
Regional identification (*z*-score)					0.0873**		0.0940
					(0.0346)		(0.0502)
*Country level*							
Net migration rate 2015 (*z*-score)			0.0074	0.2685***			
			(0.0531)	(0.0619)			
Willingness to fight for country (*z*-score)			0.0285	0.0495			
			(0.0530)	(0.0619)			
Effective democracy index 1996–2006 (*z*-score)			− 0.1996**	0.2038**			
			(0.0548)	(0.0640)			
Regional authority index (*z*-score)							
Autonomous region (1 = yes)							
*Regional level*							
Urban region (*z*-score)			− 0.0346*	0.0123			
			(0.0139)	(0.0134)			
Nationalism (völk. agg., *z*-score)							
Anti-immigrant sentiment (agg. *z*-score)							
Regional identification (agg., *z*-score)							
Constant	− 0.0880	− 0.0382	− 0.1679**	0.0487	− 0.1013**		0.0753
	(0.0621)	(0.0940)	(0.0528)	(0.0604)	(0.0383)		(0.0525)
Level 3: regions	0.0403***	0.0344***	0.0362***	0.0345***	0.0125***		0.0119***
	(0.0046)	(0.0038)	(0.0042)	(0.0038)	(0.0012)		(0.0011)
Level 2: countries	0.1060***	0.2512***	0.0664***	0.0927***	0.0321***		0.0707***
	(0.0292)	(0.0644)	(0.0188)	(0.0259)	(0.0052)		(0.0111)
Level 1: individuals	0.8882***	0.7170***	0.8747***	0.7158***	0.7339***		0.6125***
	(0.0069)	(0.0057)	(0.0068)	(0.0057)	(0.0060)		(0.0051)
− 2 log-likelihood	91233.246	81160.979	90691.616	81079.513	78119.072		70713.379
*n* regions	421	421	421	421	421		421
*n* countries	29	29	29	29	29		29
*n* individual	33,332	32,146	33,332	32,146	30,495		29,637

Sources: Cruz et al. (2018); GESIS 2015a; Hooghe et al. (2016); IOM Global Migration Data Analysis Centre (2019); Welzel (2015)

*p < 0.05

**p < 0.01

***p < 0.001

The individual control variables show that a higher age, low education and a lack of migration history are associated with the positive evaluation of patriotism. A young age, education and immigration history obviously lead to reservations about patriotism. In contrast, a higher age and immigration history foster patriotism as an attitude. Model 2, on the other hand, clearly shows the concurrent tendency of chauvinistic nationalism, i.e. the belief in the superiority of one’s own nation, and regional identification as feelings of attachment to the region of origin. Regional identification behaves similarly, only more weakly, with regard to the positive evaluation of patriotism. The context variables in the control model, however, do not show a coherent picture. The willingness to fight index is not significant for either dependent variable, while relatively ineffective democracy and rurality at the regional level are associated with a positive evaluation of patriotism. Migration does not play a role in the positive assessment of patriotism. Positive migration rates and effective democracy, on the other hand, are associated with patriotism. Table 4 shows the last two models. Model 3 focuses on the regional variables, model 4 includes all variables in the analysis.

**Table 4:** Regional identification and overall models

	Model (3)		Model (4)	
	Identification		All	
	Evaluation	Patriotism	Evaluation	Patriotism
	β/(SE)	β/(SE)	β/(SE)	β/(SE)
*Individual level*				
Sex (1 = male)	0.0195	0.0169	0.0066	0.0134
	(0.0103)	(0.0094)	(0.0100)	(0.0092)
Age (*z*-score)	0.0277***	0.0086	− 0.0132*	− 0.0207***
	(0.0055)	(0.0051)	(0.0054)	(0.0050)
Education (*z*-score)	− 0.1026***	0.0117*	− 0.0649***	0.0371***
	(0.0062)	(0.0056)	(0.0061)	(0.0056)
Immigration background (1 = no)	0.0668***	− 0.1011***	0.0122	− 0.1301***
	(0.0180)	(0.0163)	(0.0176)	(0.0162)
Nationalism (chauv.) (*z*-score)			0.3515***	0.2791***
			(0.0271)	(0.0272)
Regional identification (*z*-score)	0.1530***	0.1532*	0.0847**	0.0952***
	(0.0420)	(0.0635)	(0.0271)	(0.0272)
*Country level*				
Net migration rate 2015 (*z*-score)			− 0.0767*	0.1284***
			(0.0367)	(0.0368)
Willingness to fight for country (*z*-score)			0.0296	0.1427***
			(0.0406)	(0.0407)
Effective democracy index 1996–2006 (*z*-score)			− 0.1433***	0.2509***
			(0.0322)	(0.0322)
Regional authority index (*z*-score)	− 0.0618	0.1632*	0.0075	0.1414**
	(0.0439)	(0.0665)	(0.0435)	(0.0437)
Autonomous region (1 = yes)	0.1934*	0.1775	0.0880	0.0095
	(0.0899)	(0.1357)	(0.0609)	(0.0610)
*Regional level*				
Urban region (*z*-score)			− 0.0013	0.0005
			(0.0105)	(0.0101)
Nationalism (völk. agg., *z*-score)			0.0228	0.0516***
			(0.0152)	(0.0115)
Anti-immigrant sentiment (agg. *z*-score)			0.0269***	− 0.0324***
			(0.0063)	(0.0061)
Regional identification (agg., *z*-score)	0.0548***	0.0134	0.0241	− 0.0121
	(0.0146)	(0.0142)	(0.0134)	(0.0128)
Constant	− 0.2323***	− 0.0052	− 0.7819***	0.8690***
	(0.0554)	(0.0813)	(0.1563)	(0.1510)
Level 3: regions	0.0194***	0.0191***	0.0118***	0.0113***
	(0.0019)	(0.0018)	(0.0012)	(0.0010)
Level 2: countries	0.0477***	0.1135***	0.0187***	0.0191***
	(0.0094)	(0.0218)	(0.0032)	(0.0033)
Level 1: individuals	0.8461***	0.6824***	0.7363***	0.6123***
	(0.0067)	(0.0055)	(0.0060)	(0.0051)
-2 log-likelihood	87569.196	77992.739	78033.213	70572.351
*n* regions	421	421	421	421
*n* countries	29	29	29	29
*n* individual	32,478	31,375	30,495	29,637

Sources: Cruz et al. (2018); GESIS 2015a; Hooghe et al. (2016); IOM Global Migration Data Analysis Centre (2019); Welzel (2015)

*p < 0.05

**p < 0.01

***p < 0.001

At the individual level, regional identification exerts a more marked influence on the positive evaluation of patriotism than on patriotism itself. The two measures of regional self-determination at the country level, on the other hand, vary. While the presence of autonomous regions in a state is positively associated with the evaluation of patriotism, this is precisely not the case for the regional authority index. Here, the regression coefficients are reversed. Attachment to the region is, at the regional scale, also only positively associated with the evaluation of patriotism.

What is striking about the explanation of patriotism is the divergence between the two forms of nationalism at the regional level. Patriotism thus goes hand in hand with a rejection of *völkisch* nationalism in a region. This also gives a coherent picture. Patriotism is stronger in states with a strong democracy and high level of immigration, low regionalist tendencies but chauvinist-nationalist attitudes. Above all, a chauvinist-nationalist and regionalist attitude combined with ineffective democracy and an ethnically diverse population are associated with a positive view of patriotism. Regional identification behaves like chauvinist nationalism, only more weakly.

Empirical work on sports broadcasts, for example, reveals a significant alignment of patriotism and regional attachment. However, region is thought of as subcontinental (e.g. Chiang and Jane 2013) rather than subnational in this work. Regional attachment conceived as a reference to a continental unit then correlates with patriotism (Chiang and Jane 2013). Such data are not available for domestic regional identification, but the regression models suggest that it behaves more like nationalism. This raises the question of the pivot point, i.e. at what regional size does regional identification turn from a more nationalist affinity to a patriotic affinity? This question is not mundane, as work on forms of illiberal regional attachment clearly shows that it can undermine the foundations of international coexistence in tandem with nationalism, as exemplified by the sometimes violent political realignment of the post-Soviet world (Makarychev and Yatsyk 2018). In contrast, regional identification at the local level is more likely to be reflected in preferences for the local, such as regional food (Skallerud and Wien 2019). Here, the regression models can only establish the alignment of regionalism and nationalism at the country level.

The results of the regression models are confirmed with regard to the countervailing effects of nationalism and patriotism in work on group-based hierarchies. In these works, a correlation between nationalist attitudes and a preference for such group-based dominance structures is shown, which is precisely not found for patriotism (Osborne et al. 2017). In Germany, for example, chauvinist nationalism correlates quite well with a preference for democratic parties, whereas the *völkisch* variant is closely associated with a preference for right-wing parties (Mader et al. 2021). One possible explanation for the alignment of nationalism and regionalism in model 4 could be found at the discursive level. Using the example of Spanish nationalism, Muro and Quiroga (2005) show how regionalist sources of identity play into the shaping of nationalism by evoking nationalism as a decidedly defensive attitude towards regional identification. Spanish nationalism is thus the counterpart of Catalan and Basque regionalism and an expression of the opposition between center and periphery (Muro and Quiroga 2005), which has (co-)determined the structure of space in Europe since the Middle Ages (Stichweh 2006).

The multilevel regression models confirm hypothesis one, regional attachment at the individual level promotes a positive evaluation of patriotism and patriotism itself. Hypothesis two, on the other hand, cannot be confirmed. A dysfunctional democracy even at the level of regional decision-making power is associated with a positive evaluation of patriotism. Effective democracy is positively linked to patriotism itself. The same applies to the willingness to fight for one’s own country. This again indicates that respondents tend to associate nationalistic content with the “strong patriotic feelings” (GESIS 2015b) that were asked about in the specific question in the questionnaire. Another important factor is the symbolism of autonomous regions, which also leads to a positive assessment of patriotism. However, the effect disappears in the overall model. The stronger association of patriotism with migration figures and effective democracy makes the theoretically discussed potential of patriotism to generate collective identifications seem plausible (Erez and Laborde 2019). However, a “cosmopolitan patriotism” (Erez and Laborde 2019) would have to distinguish itself even more clearly from nationalist and regionalist attitudes, even at the regional scale.

## Conclusion

The analysis clearly shows the differentiated influence of regional identification on patriotism and its evaluation. This connection has rarely been addressed in previous research. Especially in view of the discussed increase in the importance of regional societies, e.g. with regard to the integration of ethnic minorities (Agnew 2013), there is a great need for regional research that examines regional identification on different scales relating to its changing characteristics. The regional scale level as a subnational unit is clearly gaining in importance. For example, mitigating influences of regional autonomy on anti-immigrant attitudes (Dirksmeier 2021) or, in connection with Brexit, regional variance in hostility towards migration are becoming apparent (Manow 2019). COVID-19 reveals regional differences in states with regard to support for democracy, for instance in Germany (Richter et al. 2021). Attitudes towards climate change and consequently support for climate-friendly policies can vary extremely across regions (Howe et al. 2015). Pronounced regionalist, mostly conservative parties in turn lead to support for regional industries, even if they are climate-damaging (Beer 2018).

The nation state, on the other hand, remains the essential organisational unit of the political, even if the region is empirically gaining in importance. Patriotism can be read here as a measure of attachment to this organisational unit of the state. The question of the influence of regional ties on patriotic attitudes and their evaluation is thus essential. The results of the regression models show, first of all, that openness to the world, regional support for immigration and efficient democracy are important for identification with the political organisational unit of the state. Patriotism and regional identification go hand in hand. A culture of cosmopolitanism in a region (Erez and Laborde 2019) leads to a positive attachment to the nation state. The evaluation of patriotism, on the other hand, tilts more towards the nationalist side. The significance of the regression coefficients varies across models, which indicates a fundamentally different understanding of patriotism in the case of its evaluation. Low immigration, xenophobia, nationalist attitudes, and low education lead to positive evaluations of patriotism. Regional identification has a similarly large coefficient at the individual level for evaluation of patriotism as for patriotism itself. This ultimately shows the Janus-faced nature of regional identification, which can contribute to an inclusive society as much as to a nationalist-chauvinist attitude and which has so far been overlooked in regional science.

The analysis reveals that, notwithstanding the relational and constructivist discourses, the regional scale level has an evident empirical influence on the concept of statehood. The context of the regional bond might be relevant in this case as regional identification could function as a means of political rescaling (Calzada 2018) and underpinning a specific nationalism, for example in the context of postcolonial regional movements (Söderbaum 2016). This raises the question of the influences of the regional on political and social cohesion, which can be both threatening and supportive (Dirksmeier and Göb 2021).

## Supplementary Information

Below is the link to the electronic supplementary material.

Supplementary file1 (DOCX 15 kb)

## Data Availability

The main data that support the findings of this study are available from GESIS but restrictions may apply to the availability of these data. All sources are cited in the paper.

## Code availability

SPSS with R extension (Version 3.5) and JASP 0.16.2 was used.
