# Supplementary material for: The relationship between patriotism and regional identification: a cross-country analysis
Source: Ann Reg Sci. 2022 Aug 18:1–20. Online ahead of print. doi: 10.1007/s00168-022-01167-1 (PMC9386670; doi:10.1007/s00168-022-01167-1)
Supplement: Supplementary file 1 — Supplementary file1 (DOCX 15 kb) [file 168_2022_1167_MOESM1_ESM.docx]

**Supplement**

We use maximum likelihood regression in the analysis due to the high number of context units at the regional level. Due to the relatively small sample size at the country level, Restricted Maximum Likelihood (REML) with the Kenward-Roger Approximation for optimising significance tests of fixed effects (Kenward and Roger 1997) is used as an additional robustness check. REML is being equal to ANOVA estimates in cases of equal group sizes, which yields much better estimates for small numbers of groups (Hox et al. 2018). The robustness check shows only minimal deviations of the coefficients and standard errors as well as minor changes in the covariance parameters. The results of the analysis show robustness with respect to the estimation method and significance tests for the fixed effects.

Table S1: Three-level regression models on positive evaluation of patriotism and patriotism, REML with Kenward-Rogers Approximation

|  | **Model (3)**  *Regionalism* | | **Model (4)**  *All* | |
| --- | --- | --- | --- | --- |
|  | **Evaluation**  *β/(SE)* | **Patriotism**  *β/(SE)* | **Evaluation**  *β/(SE)* | **Patriotism**  *β/(SE)* |
| *Individual level*  Sex *(1 = male)* | 0.0195 (0.0103) | 0.0169 (0.0094) | 0.0065 (0.0100) | 0.0134 (0.0092) |
| Age *(z-score)* | 0.0277*** (0.0055) | 0.0086 (0.0051) | -0.0132* (0.0054) | -0.0208*** (0.0050) |
| Education *(z-score)* | -0.1025*** (0.0062) | 0.0117* (0.0056) | -0.0649*** (0.0061) | 0.0371*** (0.0056) |
| Immigration background *(1 = no)* | 0.0668*** (0.0180) | -0.1011*** (0.0163) | 0.0124 (0.0176) | -0.1299*** (0.0162) |
| Nationalism (chauv.) *(z-score)* |  |  | 0.3514*** (0.0285) | 0.2792*** (0.0287) |
| Regionalism *(z-score)* | 0.1530*** (0.0436) | 0.1531* (0.0658) | 0.0847** (0.0285) | 0.0951*** (0.0286) |
| *Country level*  Net migration rate 2015 *(z-score)* |  |  | -0.0771* (0.0386) | 0.1287*** (0.0387) |
| Willingness to fight for country *(z-score)* |  |  | 0.0301 (0.0462) | 0.1433*** (0.0428) |
| Effective democracy index 1996–2006 *(z-score)* |  |  | -0.1429*** (0.0338) | 0.2505*** (0.0339) |
| Regional authority *index (z-score)* | -0.0617 (0.0456) | 0.1632* (0.0658) | 0.0079 (0.0457) | 0.1415** (0.0460) |
| Autonomous region *(1 = yes)* | 0.1936* (0.0932) | 0.1778 (0.1407) | 0.0853 (0.0640) | 0.0099 (0.0642) |
| *Regional level* |  |  |  |  |
| Urban region *(z-score)* |  |  | -0.0015 (0.0106) | 0.0007 (0.0101) |
| Nationalism (völk. agg., z-score) |  |  | 0.0228 (0.0154) | 0.0507*** (0.0147) |
| Anti-immigrant sentiment *(agg. z-score)* |  |  | 0.0270*** (0.0064) | -0.0328*** (0.0061) |
| Regionalism *(agg., z-score)* | 0.0552*** (0.0147) | 0.0139 (0.0142) | 0.0250 (0.0135) | -0.0110 (0.0129) |
| Constant | -0.2323*** (0.0572) | -0.0053 (0.0842) | -0.7851*** (0.1583) | 0.8772*** (0.1529) |
| Level 3: Regions | 0.0195*** (0.0019) | 0.0191*** (0.0018) | 0.0118*** (0.0012) | 0.0114*** (0.0010) |
| Level 2: Countries | 0.0515*** (0.0105) | 0.1223*** (0.0242) | 0.0210*** (0.0037) | 0.0215*** (0.0039) |
| Level 1: Individuals | 0.8462*** (0.0067) | 0.6825*** (0.0055) | 0.7337*** (0.0060) | 0.6124*** (0.0051) |
| -2 Log-Likelihood | 87622.562 | 78043.589 | 78132.980 | 70672.999 |
| n *Regions* | 421 | 421 | 421 | 421 |
| n *Countries* | 29 | 29 | 29 | 29 |
| n *Individual* | 32,478 | 31,375 | 30,495 | 29,637 |

Sources: Cruz et al. (2018); GESIS 2015a; Hooghe et al. (2016); IOM Global Migration Data Analysis Centre (2019); Welzel (2015); *p < 0.05; **p < 0.01; ***p < 0.001
